# Ocular lens morphology is influenced by ecology and metamorphosis in frogs and toads

**DOI:** 10.1098/rspb.2022.0767

**Published:** 2022-11-30

**Authors:** Amartya T. Mitra, Molly C. Womack, David J. Gower, Jeffrey W. Streicher, Brett Clark, Rayna C. Bell, Ryan K. Schott, Matthew K. Fujita, Kate N. Thomas

**Affiliations:** ^1^ Division of Biosciences, University College London, London, UK; ^2^ Department of Life Sciences, The Natural History Museum, London SW7 5BD, UK; ^3^ Imaging and Analysis Centre, The Natural History Museum, London SW7 5BD, UK; ^4^ Department of Biology, Utah State University, Logan, UT 84322, USA; ^5^ Department of Vertebrate Zoology, National Museum of Natural History, Smithsonian Institution, Washington, DC 20560-0162, USA; ^6^ Department of Herpetology, California Academy of Sciences, San Francisco, CA 94118, USA; ^7^ Department of Biology and Centre for Vision Research, York University, Toronto, Ontario, Canada; ^8^ Department of Biology, Amphibian and Reptile Diversity Research Center, The University of Texas at Arlington, Arlington, TX 76019, USA

**Keywords:** adaptation, eye, morphological evolution, sensory biology, visual ecology, CT scanning

## Abstract

The shape and relative size of an ocular lens affect the focal length of the eye, with consequences for visual acuity and sensitivity. Lenses are typically spherical in aquatic animals with camera-type eyes and axially flattened in terrestrial species to facilitate vision in optical media with different refractive indices. Frogs and toads (Amphibia: Anura) are ecologically diverse, with many species shifting from aquatic to terrestrial ecologies during metamorphosis. We quantified lens shape and relative size using 179 micro X-ray computed tomography scans of 126 biphasic anuran species and tested for correlations with life stage, environmental transitions, adult habits and adult activity patterns. Across broad phylogenetic diversity, tadpole lenses are more spherical than those of adults. Biphasic species with aquatic larvae and terrestrial adults typically undergo ontogenetic changes in lens shape, whereas species that remain aquatic as adults tend to retain more spherical lenses after metamorphosis. Further, adult lens shape is influenced by adult habit; notably, fossorial adults tend to retain spherical lenses following metamorphosis. Finally, lens size relative to eye size is smaller in aquatic and semiaquatic species than other adult ecologies. Our study demonstrates how ecology shapes visual systems, and the power of non-invasive imaging of museum specimens for studying sensory evolution.

## 1. Introduction

Shifts from aquatic to terrestrial lifestyles were likely one of the most important environmental factors driving the evolution of many aspects of vision in animals [1–3], including the key refractive components of the eye [4]. In vertebrates, these comprise the lens, a structure inside the eye, and the cornea, a layer on the front of the eye [5]. Together they focus light from a broad external field of view onto the retina at the back of the eye. Image formation is influenced by the external environment because the amount and angle of refraction that occurs when light passes between two materials (e.g. air to cornea) is dependent on the refractive indices of each material [6]. Consequently, the external environment can act as a key selective agent on refractive components, including the morphology of the ocular lens, and thus these features are expected to vary among species that differ in visual ecology.

Because their refractive index is very close to that of water, corneas have minimal refractive power in water and lenses act as the principal refractive structures in the eyes of aquatic animals [5,7,8]. To obtain a focused image underwater, aquatic animals with camera-type eyes require a powerful, spherical lens, which are found in most aquatic vertebrates such as teleost fishes, elasmobranchs (sharks and rays), agnathan fishes and marine mammals such as cetaceans and pinnipeds [5,9]. By contrast, a spherical lens in a terrestrial animal will produce excessive refractive power, causing myopia [8]. This is because corneas have a lower refractive index than air, providing them with high refractive power that typically exceeds the power of the lens [8,10]. To compensate for this, many terrestrial vertebrates have evolved flattened lenses with longer focal lengths and lower powers [5,8,11]. Alternatively, some terrestrial mammals active in low light (e.g. nocturnal opossums [5]) have relatively large (compared with the eye), spherical lenses with short focal lengths. This set-up increases visual sensitivity but simultaneously decreases acuity in spherical eyes [8]. Thus, both the shape and relative size of lenses across diverse species are predicted to be under selection to match the particular visual needs of a given species, including the medium through which vision occurs and the light levels in which they are active.

Anuran amphibians (frogs and toads) are of particular interest to explore optical adaptations, because many species recapitulate evolutionary transitions from aquatic to terrestrial environments through ontogeny. Frogs typically have biphasic life cycles in which they metamorphose from a larval tadpole stage (usually aquatic) into an adult stage (often terrestrial) [12]. Most anurans are highly visual as adults, relying on vision for prey detection, predator evasion and communication [13]. Anurans also inhabit a variety of light environments by exhibiting diverse aquatic, scansorial, semiaquatic, ground-dwelling, subfossorial and fossorial habits (lifestyles) and different diel activity patterns across species [14].

Lens shape has been studied in a few frogs and toads but remains unexplored in most of the greater than 7300 species of extant anurans. Among the species examined so far, aquatic tadpoles have spherical lenses that become axially flattened after metamorphosis in species that transition to (semi)terrestrial adults, but which remain spherical in species that remain aquatic as adults [15–18]. Although this provides evidence for adaptation to vision in different optical media, these findings are based on only five species with (semi)terrestrial adults (*Pelobates syriacus* [15], *Lithobates catesbeianus* [16], *Anaxyrus (Bufo) americanus* [17], *Pleurodema bufoninum* and *Pl. somuncurense* [18]), and only one that remains aquatic after metamorphosis (*Xenopus laevis* [16]). Lens shape is undocumented for the vast majority of anurans, and it is unknown how evolutionary history or light levels may influence changes in lens shape through ontogeny, or variability across species and life stages. For example, tadpoles of *Thoropa miliaris* are semiterrestrial with vision occurring primarily in air [19]), but have spherical lenses that begin flattening only in late larval stages; adult lenses in this species have not been examined [20]. Further, we might expect species that are active in low light (e.g. nocturnal and/or burrowing species) to have relatively large, spherical lenses with short focal lengths to maximize sensitivity, and this has not been explored in anurans. The goal of this study was to investigate macroecological patterns in lens shape and relative size across anuran species and life stages.

We used high-resolution micro X-ray computed tomography (microCT) to examine 126 species of frogs and toads across 41 of the 58 currently recognized families [21] to better understand the ecological, ontogenetic and phylogenetic determinants of lens morphology. Based on expectations for optimal optics in different optical media and light levels, we tested the following hypotheses: (i) tadpoles have more spherical lenses than adult anurans; (ii) lens shape changes across metamorphosis for species that occupy aquatic environments as larvae and terrestrial environments as adults; and (iii) adult lens shape and relative size correlate with habit and activity pattern.

## 2. Methods

### (a) Specimen processing and micro X-ray computed tomography imaging

**Figure 1.**
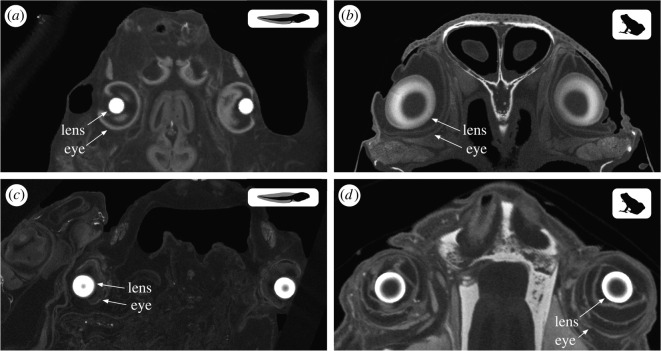
MicroCT scan slice images of larvae (tadpole silhouette) and adults (frog silhouette) illustrating lens shape across metamorphosis for two anuran species with different adult habits. (*a*) The *Schismaderma carens* tadpole (aquatic) has spherical lenses that (*b*) flatten axially in the ground-dwelling (terrestrial) adult. (*c*) Spherical lenses of the *Xenopus victorianus* tadpole are (*d*) retained in the aquatic adult.

Anurans generally have firm lenses, with accommodation achieved by moving the lens closer to and further from the retina, rather than by deformation as in mammals [22]. Thus, lens shape measured in preserved specimens should reflect shape in life, making museum specimens amenable for this study. We used microCT scans to non-invasively obtain three-dimensional (3D) size and shape data for lenses in whole, fluid-preserved museum specimens (figure 1). These data were from a mixture of unstained (*n* = 98) and iodine-stained (*n* = 81) specimens, because scans were compiled from several ongoing and published studies. Iodine staining is used to enhance X-ray absorption, improving image contrast. Scans were made at the Smithsonian National Museum of Natural History (NMNH, Washington, DC, USA; *n* = 46), the Natural History Museum, London (BMNH, London, UK; *n* = 40), the University of Florida Nanoscale Research Facility (UF, Gainesville, FL, USA; *n* = 46) and the Cornell Institute of Biotechnology (CIB, Ithaca, NY, USA; *n =* 1), or obtained from MorphoSource (https://www.morphosource.org; *n* = 46). See electronic supplementary material for additional details of specimen microCT scanning, staining and preparation (electronic supplementary material, appendix A).

### (b) Taxon sampling and ecological classification

We measured lens shape in 179 anuran specimens (128 adults and 51 tadpoles) from 126 species and 41 families [21]. We generated matched data for tadpole and adult stages for 45 of these species from 24 families (figure 3). These species were all biphasic with aquatic tadpoles, and the primary adult environment for each was categorized as either aquatic or terrestrial. Among species with adult lens data (*n* = 121), each species was scored for two discrete ecological categories following [23]: (i) the predominant adult habit is aquatic, semiaquatic, scansorial, ground-dwelling, subfossorial or fossorial, and (ii) the predominant adult activity pattern is nocturnal or non-nocturnal, the latter including diurnal, arrhythmic and cathemeral or crepuscular species. Species lacking data for a particular ecological trait were excluded from analyses of that trait. See electronic supplementary material for references used in trait scoring (electronic supplementary material, appendix B).

### (c) Segmentation and three-dimensional shape metrics of lenses

Lenses were segmented into 3D voxel models using Avizo v. 2019.1 (https://www.thermofisher.com/avizo). Segmentation was performed manually for unstained scans and by using the automated magic wand tool (region growing) for stained scans using specimen-specific thresholds. The software was used to generate four 3D shape metrics for each lens that captured different axes of deviation from a perfect spherical shape: anisotropy, flatness, elongation and sphericity. Anisotropy, flatness and elongation were calculated by analysing ratios of eigenvalues of the covariance matrix. In brief, a perfect sphere has an anisotropy of 0, flatness of 1 and elongation of 1. Sphericity was calculated as the ratio of the surface area of a perfect sphere with the same volume as the lens to the measured surface area of the lens. Perfect spheres have a sphericity value of 1. See electronic supplementary material for additional details on the calculation of these metrics (electronic supplementary material, appendix A).

### (d) Cross-sectional two-dimensional eye and lens measurements

While anuran lenses were generally undeformed and clearly discernible in scans, the soft tissues of eyes varied more in scan contrast and physical condition. Adult eye and lens diameters were measured for 81 anuran species with intact and adequately imaged eyes. Eye measurements were not measured for tadpoles because, although their lenses consistently appeared undamaged, the soft tissues of their eyes were often disfigured, presumably during preservation or storage. Although the most optically relevant measures of eye and lens size are axial lengths measured along the anterior–posterior axis of the eye, we were unable to clearly delineate the posterior boundary of the eyes in many scans. Thus, we instead measured transverse eye and lens diameters along the naso-temporal axis (as defined in [24]) of each eye. These two-dimensional (2D) measurements were obtained from scans in Avizo by positioning a horizontal plane (as defined in [24]) bisecting the 3D rendering of the eye and lens and so that it yielded the maximum transverse eye and lens diameters. Measurements for both eyes (usually obtained from two different slices) were averaged for each specimen and across specimens for each species.

### (e) Statistical analyses and data validation

Analyses were performed in R v. 4.0.3 [25] via RStudio v. 1.2.5033 [26]. Prior to testing our hypotheses (below), we assessed measurement repeatability and ran data-validation analyses to confirm that error in scan measurements and multicollinearity of shape measurements would not mislead our analytical results (electronic supplementary material, appendix C, tables S1–S4, figures S1 and S2). This led us to exclude flatness from statistical analyses, because it was highly correlated with anisotropy (electronic supplementary material, figure S3 and table S3). For all analyses incorporating phylogeny, we used a published amphibian phylogeny [27] pruned in ape v. 5.5 [28] to the species in our datasets.

### (f) Do tadpoles have more spherical lenses than adult anurans?

We first examined whether tadpole lens shape differed from adult lens shape across all species sampled (121 species with adult data; 50 species with tadpole data). We did this (i) with a principal component analysis (PCA) of species means for 3D lens-shape metrics (anisotropy, elongation and sphericity) using the base package stats v. 4.2.1 in R and (ii) with unpaired Student's *t*-tests for differences across life stages for each lens-shape metric. Finally, to understand how patterns in lens shape may be structured by evolutionary history, we estimated phylogenetic signal (*K*_mult_) in all three lens-shape metrics together for tadpoles (*n* = 50) and adults (*n* = 121) using the physignal function in geomorph v. 4.0.0 [29], which estimates the multivariate version of the *K*-statistic (*K*_mult_) [30].

### (g) Does lens shape change across metamorphosis for species that occupy aquatic environments as larvae and terrestrial environments as adults?

Next, we tested whether changes in lens shape across metamorphosis are greater for species with aquatic tadpoles transitioning to terrestrial adults than for biphasic species that remain aquatic as adults. We examined species for which we had measurements for both tadpole and adult stages (*n* = 45). To understand how the change in lens shape across metamorphosis may be structured by evolutionary history, we estimated multivariate phylogenetic signal in the differences between adult and tadpole lens-shape metrics (anisotropy, sphericity and elongation) together using the physignal function in the geomorph package (see above). We then used univariate phylogenetic least-squares (PGLS) models in caper v. 1.0.1 [31] to test whether ontogenetic transitions from aquatic to terrestrial environments had an effect on the difference between tadpole and adult species means for each 3D shape metric separately. In these analyses, we estimated phylogenetic signal (*λ*) in residual error simultaneously with the regression parameters, using maximum-likelihood [32].

### (h) Does adult lens shape or relative size correlate with habit or activity pattern?

Lastly, we tested whether adult lens shape or relative size are correlated with adult ecology (habit or activity pattern). For shape, we fit a series of univariate PGLS models in caper to test whether each adult lens-shape metric (anisotropy, elongation or sphericity) was correlated with each ecological trait (habit or activity pattern). To investigate lens size relative to eye size, we ran two PGLS regressions in caper to test for associations between lens diameter and (i) habit (*n* = 80 species), and (ii) activity pattern (*n* = 73 species), both with eye diameter as a covariate.

## 3. Results

### (a) Tadpoles have more spherical lenses than adult anurans

**Figure 2.**
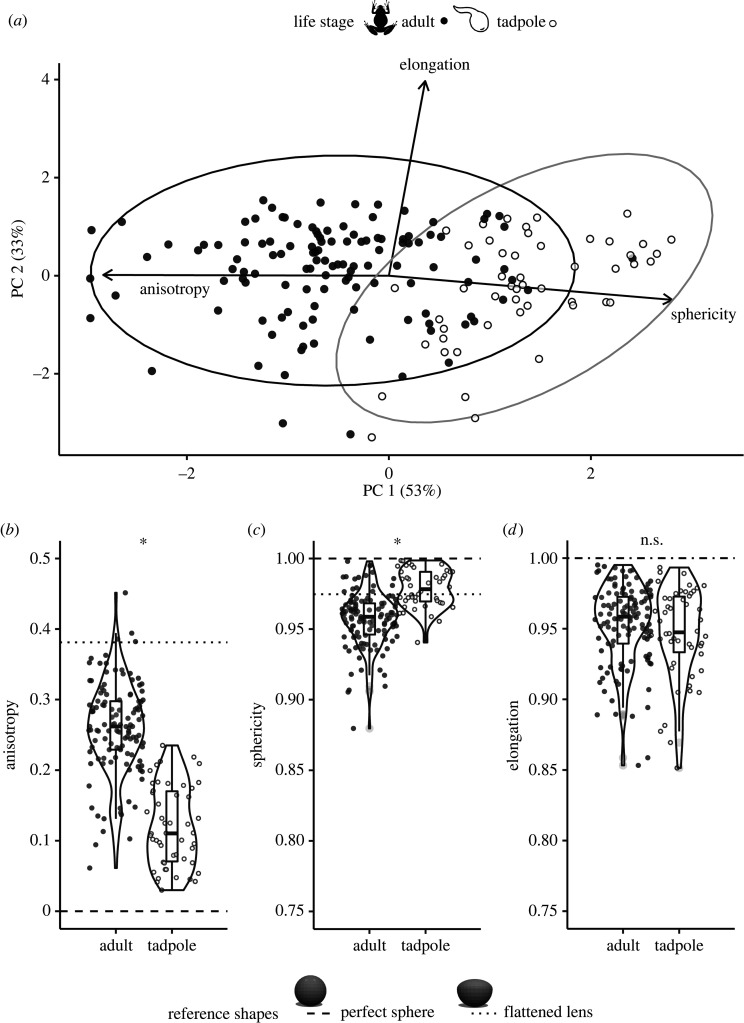
Variation in ocular lens shape across anuran adults (121 species) and tadpoles (50 species). (*a*) Principal component analysis (PCA) of metrics used to quantify 3D lens shape (anisotropy, sphericity and elongation), with ellipses showing the 95% confidence level for a multivariate normal distribution in adults (black) and tadpoles (grey). Arrows indicate direction and weighting of vectors representing the three shape metrics analysed. Adults (filled circles) and tadpoles (unfilled circles) are partially separated along principal component (PC) 1. (*b–d*) Violin plots depict data for each shape metric across life stages. Tadpoles and adults differ significantly in lens (*b*) anisotropy and (*c*) sphericity, but not (*d*) elongation. Note that the *y*-axes differ on *b–d*; all three metrics are proportional values theoretically ranging from 0 to 1, but axes have been trimmed to match the range of data. The dashed line indicates the value of each shape metric for a perfect sphere, and the dotted line the value for a sphere flattened anteriorly in rough approximation of a typical terrestrial adult frog lens.

Tadpoles and adults showed differences in lens shape that were best captured by measurements of anisotropy and sphericity (figure 2). In a PCA of shape metrics, total variance among species means for tadpoles and adults was accounted for mostly by PC1 (52.2%) and PC2 (33.3%) and less so by PC3 (14.5%). PC1 had large-magnitude PC loadings for anisotropy (−0.71) and sphericity (0.70), while PC2 had a larger PC loading for elongation (−0.99). Tadpoles and adults fell into partially distinct clusters based on PC1 values (figure 2*a*). Lens shape across species and life stages varied most in anisotropy and least in elongation (figure 2*b–d*).

Overall, anuran tadpoles had more spherical lenses than adults, and the greater flattening of adult lenses was captured by measures of anisotropy and sphericity. Adult anurans (*n* = 121 species), on average, had lenses that were more anisotropic (*t*-test: d.f. = 105.6, *t* = 14.1, *p* < 0.001) and less spherical (*t*-test: d.f. = 116.9, *t* = −8.93, *p* < 0.001) than tadpoles (*n* = 50 species; figure 2). Lens elongation did not differ significantly between adults and tadpoles (*t*-test: d.f. = 80.4, *t* = 1.20, *p* = 0.24). Phylogenetic signal (*K*_mult_) in multivariate lens shape was 0.44 in adults (*p* = 0.001, *n* = 121), but was not significant in tadpoles (*K*_mult_ = 0.46, *p* = 0.41, *n* = 50).

### (b) Lens shape changes across metamorphosis for species that occupy aquatic environments as larvae and terrestrial environments as adults

**Figure 3.**
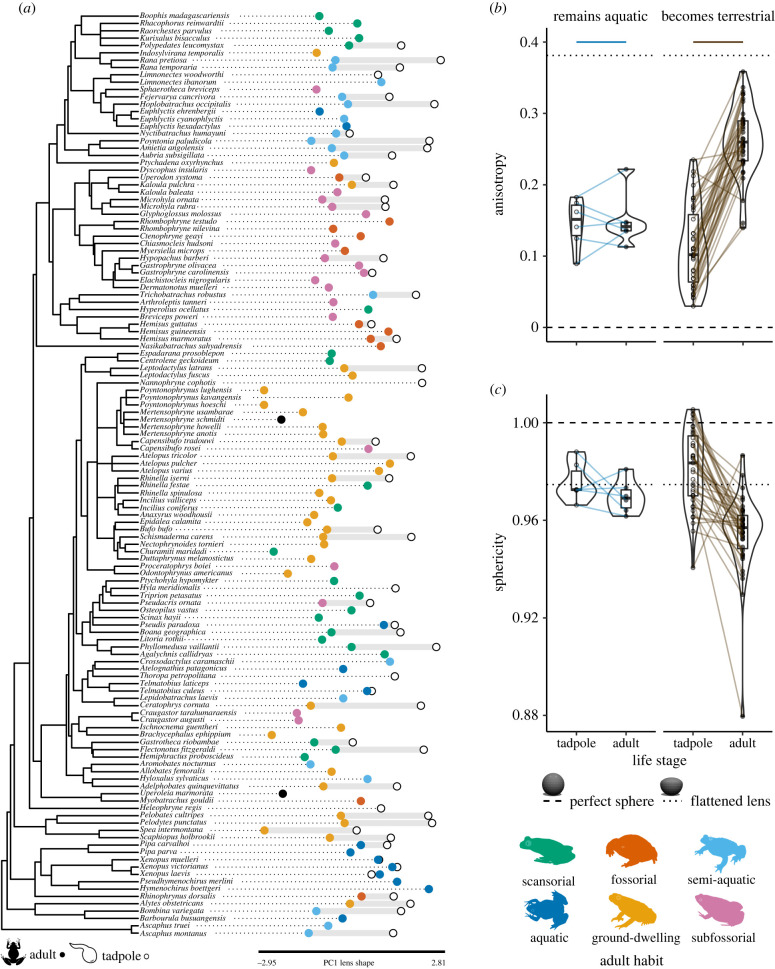
Anuran lens shape across life stages, environments and adult habits. (*a*) Anuran phylogeny of 126 species across 41 families (adapted from [27]). Variation in lens shape is depicted with PC1 scores and the difference between PC1 scores of tadpoles (unfilled circles) and adults (filled circles, coloured by adult habit or black if habit unknown). (*b*) Anisotropy and (*c*) sphericity within species (*n* = 45) change significantly less in species with aquatic tadpoles that metamorphose into aquatic adults (blue) than for aquatic tadpoles that metamorphose into terrestrial adults (brown). Tadpole lens data are shown with unfilled circles and adult data with filled circles, with lines connecting data within each species. The dashed line indicates the value of each shape metric for a perfect sphere, and the dotted line the value for a sphere flattened anteriorly in rough approximation of a typical terrestrial adult frog lens.

Species with aquatic larvae that remain aquatic as adults maintain spherical lenses across ontogeny, while those that transition to terrestrial environments typically undergo changes at metamorphosis that result in flatter lenses. This change in shape was captured by measures of anisotropy and sphericity. Post-metamorphic environment had a significant effect on the ontogenetic change in lens anisotropy (PGLS: *F*_1,43_ = 19.7, *λ* = 0.44, *R*^2^_adj_ = 0.30, *p* < 0.001) and sphericity (PGLS: *F*_1,43_ = 4.61, *λ* = 0.25, *R*^2^_adj_ = 0.08, *p* = 0.04) within species (*n* = 45). Species transitioning from aquatic larval to terrestrial adult environments showed a marked increase in lens anisotropy (PGLS: *β* = 0.15, s.e. = 0.03) and a small decrease in sphericity (PGLS: *β* = −0.03, s.e. = 0.01) across life stages, while those that remain aquatic showed little increase in anisotropy (PGLS: *β* = 0.01, s.e. = 0.03) or decrease in sphericity (PGLS: *β* = −0.01, s.e. = 0.01). Elongation did not differ significantly across post-metamorphic environments (PGLS: *F*_1,43_ < 0.001, *λ* = 0, *R*^2^_adj_ = −0.02, *p* > 0.99). Phylogenetic signal (*K*_mult_) in the ontogenetic change in multivariate lens shape (difference between tadpole and adult anisotropy, sphericity and elongation) was 0.61 (*p* = 0.01, *n* = 45, figure 3).

### (c) Lens shape and relative size correlate with adult habit but not activity period

**Figure 4.**
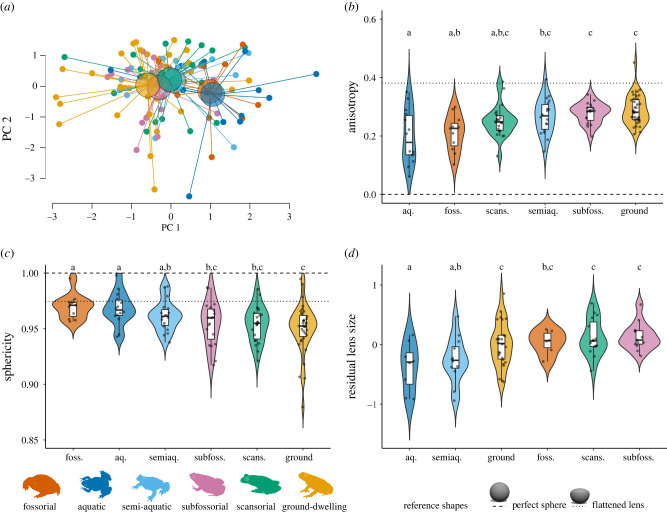
Adult anuran lens shape (*n* = 119) and relative size (*n* = 81) across species with different adult habits. (*a*) PCA centroids show multivariate adult lens shape across habits. Violin and box plots are ordered by mean and show lens (*b*) anisotropy, (*c*) sphericity and (*d*) relative size (residuals of a PGLS of lens diameter versus eye diameter) across adult habits. Letters above violin plots (a, b or c) indicate groups that are not significantly different from one another in multiple pairwise comparisons. The dashed line indicates the value of each shape metric for a perfect sphere, and the dotted line the value for a sphere flattened anteriorly in rough approximation of a typical terrestrial adult frog lens.

Adult anurans with aquatic and fossorial habits had more spherical lenses than those that are semiaquatic, subfossorial, ground-dwelling or scansorial, but metrics of lens shape did not differ among species active during different times of day (figure 4; electronic supplementary material, figure S6). Adult habit had a significant effect on species means (*n* = 119) for anisotropy (PGLS: *F*_5,113_ = 3.01, *λ* = 0.45, *R*^2^_adj_ = 0.08, *p* = 0.01) and sphericity (PGLS: *F*_5,113_ = 3.52, *λ* = 0, *R*^2^_adj_ = 0.10, *p* = 0.01), but not elongation (PGLS: *F*_5,113_ = 0.50, *λ* = 0, *R*^2^_adj_ = −0.02, *p* = 0.78; electronic supplementary material, figure S5). Pairwise comparisons showed that these differences were driven by lower anisotropy and higher sphericity in the lenses of aquatic and fossorial species (electronic supplementary material, tables S5 and S6). Adult activity pattern had no effect on species means (*n* = 107) for lens anisotropy (PGLS: *F*_1,105_ = 0.92, *λ* = 0.82, *R*^2^_adj_ = 0.00, *p* = 0.34), sphericity (PGLS: *F*_1,105_ = 0.01, *λ* = 0, *R*^2^_adj_ = −0.01, *p* = 0.94), or elongation (PGLS: *F*_1,105_ = 0.01, *λ* = 0, *R*^2^_adj_ = −0.01, *p* = 0.92).

Adult anurans with aquatic and semiaquatic habits had smaller lenses relative to their eyes than species that are fossorial, subfossorial, ground-dwelling or scansorial, but relative lens size did not differ among species active during different times of day (figure 4*d*; electronic supplementary material, figure S6d). Lens size was highly correlated with eye size but also significantly associated with adult habit. A model of lens size versus eye size with adult habit as a covariate (PGLS: *F*_6,73_ = 198.5, *R*^2^_adj_ = 0.94, *p* < 0.001) indicated that lens size relative to eye size differed significantly among species (*n* = 80) with different adult habits (*F* = 5.65, d.f. = 5, *p* < 0.001). This pattern was primarily driven by the relatively small lenses of aquatic and semiaquatic species (electronic supplementary material, table S7). A separate model including activity pattern as a covariate (PGLS: *F*_3,69_ = 269.4, *R*^2^_adj_ = 0.92, *p* < 0.001) indicated that relative lens size does not differ among species (*n* = 73) with different activity patterns (*F* = 0.64, d.f. = 2, *p* = 0.53).

## 4. Discussion

### (a) The spherical lenses of aquatic tadpoles

Most biphasic frogs and toads have an aquatic tadpole stage with eyes that view through water and a more terrestrial adult stage with eyes that view primarily through air. This change in optical medium should place differing pressures on the refractive components of the eye, such as the lens. We found that indeed, the lenses of aquatic tadpoles are distinct in shape from those of adults and tend to be more spherical, which would give them higher refractive powers ideal for focusing underwater where the cornea lacks power (figures 1 and 2) [33].

Interestingly, we found notable variation in lens shape among aquatic tadpoles, which may be explained in part by variation in larval age. Vertebrate lenses grow by the continual addition of fibres in concentric layers [34]. Because lens shape changes gradually through growth, and because tadpoles sampled in this study were of unknown, varying developmental stages, specimens closer to metamorphosis may have had less spherical lenses (at least for species that are terrestrial as adults). Variation in tadpole lens shape may also result from differences in the rate of change in lens shape during metamorphosis, which differs in three anuran species examined so far [16,17] and may be correlated to tadpole feeding habits. For example, carnivorous tadpoles of *Lithobates catesbeianus* undergo flattening of the lens during a short period just before they move onto land, while change in lens shape is more gradual in herbivorous, presumably less visually dependent tadpoles of *Pelobates syriacus* [16].

### (b) Lens shape across aquatic to terrestrial transitions

Within species, the change in lens shape across metamorphosis corresponds to the optical medium through which adult vision occurs (figures 1 and 3). In biphasic anurans that remain aquatic after metamorphosis, adult lenses were usually spherical and resembled larval lenses in shape. In those with terrestrial adults, the spherical tadpole lens generally becomes a partially flattened ellipsoid after metamorphosis, which is consistent with previous work on five anuran species with (semi)terrestrial adult life stages [15–18].

All the species included in our study have aquatic larvae. As such, we may not have captured the full extent of variation in larval lens shape because some anurans have partially or fully terrestrial development; specifically those species with either semiterrestrial tadpoles (e.g. *Cycloramphus*) or direct development (i.e. no larval stage) [12,35–37]. Although we might predict that tadpoles with terrestrial or semiterrestrial habits should have flatter, less spherical lenses than aquatic tadpoles (similar to lenses of terrestrial adults), the previously mentioned example of *Thoropa miliaris* does not fit the prediction, with their semiterrestrial tadpoles showing spherical lenses late in development [20]. Clearly, further investigation is needed to understand how terrestrial habits influence lens shape in anuran larvae. Relevant insights may be obtained from non-amphibian species, such as mudskippers, teleost fishes with aquatic larvae that develop into semiterrestrial adults with flattened lenses [38].

### (c) Adult lens shape across habits and activity periods

Although most terrestrial vertebrates adapt to vision through air by flattening their lenses to compensate for the refractive power of their corneas, there are other strategies to achieve focus with a more spherical lens on land. One option is to flatten the cornea to minimize its power in air, which can be a good strategy for species that need to use their eyes in both air and water [1]. Another is to leave the cornea unchanged but to increase the size of the lens relative to the eye to accommodate the shorter focal length of a powerful refractive system, which can maximize sensitivity for species active in dim light [1]. We found that both lens shape and relative size across adult frogs and toads correlated with species habits (figure 4), but neither shape nor size differed across activity patterns.

Across our complete dataset, fully aquatic adult frogs typically had spherical lenses, whereas most terrestrial adults (ground-dwelling, scansorial, semiaquatic, subfossorial) had flattened lenses, consistent with our findings using a smaller dataset of paired tadpole–adult life stages. These differences in lens shape with respect to species habits likely result from differences in the refractive power of the cornea in air versus water as described above. Aquatic adults also had smaller lenses relative to their eye sizes than species with primarily terrestrial habits. Previous work on lens development across life stages of the toad *Bufo bufo* found that aquatic tadpoles have small spherical lenses relative to their eyes, with a large distance between the lens and retina, similar to many teleost fishes. The lens increases in relative size, flattens anteriorly, and moves closer to the retina during metamorphosis into a terrestrial toadlet [17]. These changes are also seen during metamorphosis in the toad *Pelobates syriacus* and the salamander *Salamandra salamandra* [15,39]. Thus, the eyes of anurans that remain aquatic after metamorphosis maintain not only the spherical shape of larval lenses, but also the relative size and, likely, the focal length. Other recent work has shown that amphibian species with aquatic adults are more likely to retain the circular pupil shape of tadpoles than to exhibit the wide range of pupil shapes found among terrestrial adults [40,41]. Together, these findings suggest that the visual systems of aquatic adult anurans do not change dramatically across metamorphosis.

Semiaquatic anurans present an interesting case because they most commonly view through air but may also spend time viewing underwater. Optimum focusing can be a challenge for animals that frequently transition between optical media [42]. Accommodation, the adjustment of optical power for focusing on objects at different distances, can change focal length to some degree but cannot generally compensate fully for the large refractive differences between air and water [33]. We might predict that to cope with this, semiaquatic frogs would have spherical lenses paired with flattened corneas as seen in some other amphibious animals (e.g. seals and sea lions; some diving birds) [5,8,39,42–44]. Instead, we found that semiaquatic adults typically have flattened lenses, like most terrestrial frogs, which suggests that semiaquatic frogs may generally depend more on visual tasks in air than in water. Like aquatic frogs, however, semiaquatic adults had smaller lenses relative to their eye sizes than did more terrestrial species. The functional consequences of this are unclear, and further exploration of the refractive components of eyes, including the corneas, of semiaquatic frogs may yield new insights.

A surprising outcome of our study was that fossorial adults, despite being terrestrial, have spherical lenses similar to those of aquatic species. Given their spherical lenses and the refractive power of corneas in air, adult fossorial frogs may be myopic (near sighted) after transitioning to life underground. This could quite possibly be adaptive, because as burrowing animals foraging in small spaces underground, most of what they look at should be near to their eyes. Myopia in teleost fishes has been similarly proposed to prioritize viewing of nearby objects [5,33].

Spherical lenses in terrestrial animals can also increase visual sensitivity when paired with a wide aperture (pupil) and relatively small eye to fit a shorter focal length [8]. We found that fossorial adult anurans do have relatively large lenses for their eyes, in contrast to aquatic species. Although pupil sizes of fossorial frogs in our study have not all been quantified, the pupils of fossorial terrestrial frogs tend to be circular rather than horizontally elongated as is more common in other terrestrial groups, and thus fossorial frogs may have limited pupil constriction [41]. It is possible that the large, spherical lenses of fossorial frogs serve to increase their sensitivity in dimly lit conditions underground. Alternatively, fossorial species may have experienced relaxed selection pressure to change lens shape during metamorphosis because fossorial adults rely less on acute vision underground, as may also be demonstrated by their generally reduced investment in eye size [23]. Interestingly, some predominantly fossorial species emerge aboveground for breeding, which typically takes place in water where spherical lenses are beneficial (e.g. *Rhinophrynus dorsalis* [45]).

The association between spherical lenses and fossorial habits in frogs is distinct from what has been observed in some fossorial mammals such as the South American armadillo (*Chaetophractus villosus*), which appears to have flattened lenses [46]. However, previous work has shown that fossorial adult caecilian amphibians (Gymnophonia) with aquatic larvae have only slightly flattened lenses as adults [47]. Fossorial snakes have spherical lenses, but seemingly so do all other snakes across diverse aquatic, ground-dwelling and arboreal lifestyles [5]. In snakes, the retention of spherical lenses has been explained by phylogenetic conservatism, with the ancestral snake adapted to either extremely fossorial (promoting evolutionary degeneration of the eye) or highly aquatic habits [5,33,48]. Studies of corneal shape, focal lengths and visual capability are necessary to explore the functions of spherical lenses in fossorial frogs. More broadly, we suggest that the optical consequences of variation in lens shape among fossorial vertebrates warrants further attention.

Because a relatively large, spherical lens with a short focal length can increase sensitivity, we predicted that nocturnal frogs and toads active in low light might also exhibit these traits. The lenses of nocturnal vertebrates (e.g. owls and galagos) are often large relative to eye size, while those of arrhythmic species (e.g. dogs) are intermediate sized, and those of diurnal species (e.g. pigeons and humans) are relatively small [1,5,8]. We did not, however, find evidence for this pattern in adult anurans. Nocturnality was most likely the activity pattern of the common anuran ancestor, and most extant species retain this trait [14]. Thus, the typical adult anuran eye may contain a relatively large lens regardless of activity pattern because of this evolutionary history. However, it is important to note that our measurements of lens diameter were transverse rather than axial, and if the ratio of these lens measurements differ across activity patterns then we may not have captured important differences in focal lengths. We find this unlikely, however, because measures of 3D lens shape also did not differ across activity patterns.

Overall, we found strong evidence that lens morphology corresponds to ecology in adult anurans, though we did find high variation across species within ecological categories. Some of this may be due to the difficulty of scoring species for discrete ecological categories, which may not always be an accurate representation of their visual ecology. For instance, although adults of *Barbourula busuangensis* were scored as aquatic because they rarely leave the water, this species has been found primarily at the surface of the water with emergent eyes and nostrils [49]. *Barbourula busuangensis* has less spherical (higher anisotropy and lower sphericity) lenses than those of pipids, such as *Xenopus*, which are more active underwater as adults [50]. Likewise, the burrowing *Scaphiopus holbrooki* aestivates below ground but was scored as ground-dwelling based on its predominant habits when active (e.g. feeding and breeding). Yet, despite being visually active above ground, this species has spherical lenses with anisotropy and sphericity measurements that are closer to those of more fossorially active species. Further studies of variability in lens shape and relative lens size will promote a more complete understanding of the interplay of optical medium and light condition on the evolution of visual systems in vertebrates and may inform predictions about the unknown ecology and behaviours of some species based on lens morphology.

### (d) The use of CT-scanned museum specimens for studying ocular morphology

Studies of vertebrate lens morphology have typically employed methods limited to 2D observations, such as freeze-sectioning [16] and classical histology [5,51]. Our study (also see [20]) demonstrates that microCT scanning is an effective tool to study both 2D and 3D aspects of ocular morphology, particularly because lenses are typically undamaged and can be imaged in fluid-preserved museum specimens. This method of non-invasive data collection can also facilitate extensive taxon sampling, including from rare or extinct species. We show that 3D shape metrics such as anisotropy, flatness and sphericity, which have previously been used to study particle symmetry or porosity in material science [52], can be used for quantitative biological studies.

## 5. Conclusion

This examination of lens shape and size among anurans demonstrates the importance of optical medium and ecology in shaping anuran lens morphology across broad phylogenetic and ecological diversity. Aquatic tadpoles have spherical lenses that, in species with terrestrial adult habits, typically flatten during metamorphosis. By contrast, species with aquatic adult habits tend to retain relatively small, spherical lenses as adults that resemble larval lenses. A terrestrial exception is found in fossorial adults, which have relatively large, spherical lenses that may be adapted to foraging in small, dimly lit spaces. Activity pattern does not affect lens shape or relative size in adult anurans. The results and methodology presented here can be applied to the study of visual ecology and lens morphology in other vertebrate groups.

## Data Availability

Code to reproduce all analyses and data figures can be found in the GitHub repository: https://github.com/knthomas/frog-lens-shape. CT scan data are available on MorphoSource (https://morphosource.org; Project IDs: 000446604, 000448159 and 00000C967). Morphological measurements and ecological trait scores for all sampled species are available from the Dryad Digital Repository: https://doi.org/10.5061/dryad.4mw6m90d7 [53]. Additional analyses and results supporting this study can be found in the electronic supplementary material [54].
